# Genome Sequence of Marine Bacterium *Idiomarina* sp. Strain 28-8, Isolated from Korean Ark Shells

**DOI:** 10.1128/genomeA.00772-13

**Published:** 2013-10-03

**Authors:** Woo-Jin Kim, Young-Ok Kim, Dong-Gyun Kim, Bo-Hye Nam, Hee Jeong Kong, Hyungtaek Jung, Sang-Jun Lee, Dong-Wook Kim, Dae-Soo Kim, Sung-Hwa Chae

**Affiliations:** Biotechnology Research Division, National Fisheries Research and Development Institute (NFRDI), Busan, Republic of Koreaa; Human Derived Material Center, Korea Research Institute of Bioscience and Biotechnology (KRIBB), Daejeon, Republic of Koreab; Research Institute of GnCBIO Co., Ltd., Daejeon, Republic of Koreac

## Abstract

*Idiomarina* sp. strain 28-8 is an aerobic, Gram-negative, flagellar bacterium isolated from the bodies of ark shells (*Scapharca broughtonii*) collected from underwater sediments in Gangjin Bay, South Korea. Here, we present the draft genome sequence of *Idiomarina* sp. 28-8 (2,971,606 bp, with a G+C content of 46.9%), containing 2,795 putative coding sequences.

## GENOME ANNOUNCEMENT

The genus *Idiomarina*, in the family *Alteromonadaceae*, was first proposed by Ivanova et al. (1) to accommodate two marine bacteria: *Idiomarina abyssalis* and *Idiomarina zobellii*. *Idiomarina* species are Gram-negative, aerobic, flagellar bacteria, which have been isolated from seawater, including oceanic water, coastal sediments, and submarine hyperthermal fluids (2). *Idiomarina* sp. strain 28-8 was isolated from the bodies of ark shells (*Scapharca broughtonii*) collected from underwater sediments in Gangjin and Jinhae Bay, South Korea. This strain was cultured at 22°C in marine medium and produced proteolytic enzymes. This strain also had alpha- and beta-hemolytic activities. A phylogenetic analysis of 16S rRNA genes indicated that it was most closely related to the genus *Idiomarina*, with the greatest similarity to *Idiomarina loihiensis* (99.7%), followed by *Idiomarina ramblicola* (99.03%) and *Idiomarina abyssalis* (98.84%). Therefore, this strain was identified as *Idiomarina* sp. 28-8. Genomic DNA was extracted from the cultured bacteria using the alkaline lysis method (3). We sequenced the genome of this species according to the Genomes OnLine Database (GOLD) (4) because it had not been sequenced at the time our sequencing project began. We report here the genome sequence of *Idiomarina* sp. 28-8, obtained using a whole-genome shotgun strategy (5) with a Roche 454 GS (FLX Titanium) pyrosequencing system (768,097 reads totaling ~215.7 Mb, with ~28.9-fold coverage of the genome) (6). Pyrosequencing was processed using Roche software, according to the manufacturer’s instructions. All of the paired reads were assembled using the Newbler assembler 2.6 (454 Life Sciences), which generated 110 contigs (accession no. BANL01000001 to BANL01000110), and the assembly falls into 9 scaffolds (accession no. DF266789 to DF266797). The predicted proteins were annotated using the Basic Local Alignment Search Tool (BLAST) (7) and the Rapid Annotations using Subsystems Technology (RAST) server (8). In addition, open reading frame (ORF) prediction was performed using Cd-hit software, which searched the contigs against the Glimmer 3.02 modeling software package and GeneMark version 2.5 (9), tRNAscan-SE 1.21 (10), RNAmmer 1.2 (11), and Clusters of Orthologous Groups (COG) (12) databases to annotate the gene descriptions.

The *Idiomarina* sp. 28-8 draft genome includes 2,971,606 bp and comprises 2,795 predicted coding sequences (CDSs), with a G+C content of 46.9%. There are single predicted copies of the 5S, 16S, and 23S rRNA genes and 49 predicted tRNAs. The genome contains representatives of 423 subsystems, and we used this information to reconstruct the metabolic network, which was determined using the RAST server. A distinguishing subsystem feature is the absence of genes corresponding to ATP-dependent RNA helicase, biopolymer transport protein, signal transduction histidine, chemotaxis protein, dihydrolipoamide acetyltransferase, DNA polymerase, outer membrane protein, DNA-binding protein, and flagellar biosynthesis protein. The CDSs annotated by the COG database were classified into 12 major categories (R, S, E, L, M, P, C, K, T, O, H, and I) from among the 45 COG groups. The enzymes identified included rRNA methyltransferase (EC 2.1.1.-), DNA polymerase (EC 2.7.7.7), tRNA pseudouridine synthase (EC 5.4.99.12), and sensor histidine kinase (EC 2.7.13.3).

### Nucleotide sequence accession numbers.

The draft genome sequence of *Idiomaria* sp. 28-8 is available in GenBank under the accession no. BANL00000000 (DF266789 to DF266797). The version described in this paper is version BANL01000000.
